# Improvements in technology and the expanding role of time-driven, activity-based costing to increase value in healthcare provider organizations: a literature review

**DOI:** 10.3389/fphar.2024.1345842

**Published:** 2024-05-22

**Authors:** Ana Paula Beck Da Silva Etges, Porter Jones, Harry Liu, Xiaoran Zhang, Derek Haas

**Affiliations:** ^1^ PEV Healthcare Consulting, São Paulo, Brazil; ^2^ Avant-garde Health, Boston, MA, United States; ^3^ Programa de Pós-graduação em Epidemiologia da Escola de Medicina da Universidade Federal do Rio Grande do Sul, Porto Alegre, RS, Brazil

**Keywords:** time-driven activity-based costing, TDABC, microcosting, technology, digital health

## Abstract

**Objective:**

This study evaluated the influence of technology on accurately measuring costs using time-driven activity-based costing (TDABC) in healthcare provider organizations by identifying the most recent scientific evidence of how it contributed to increasing the value of surgical care.

**Methods:**

This is a literature-based analysis that mainly used two data sources: first, the most recent systematic reviews that specifically evaluated TDABC studies in the surgical field and, second, all articles that mentioned the use of CareMeasurement (CM) software to implement TDABC, which started to be published after the publication of the systematic review. The articles from the systematic review were grouped as manually performed TDABC, while those using CM were grouped as technology-based studies of TDABC implementations. The analyses focused on evaluating the impact of using technology to apply TDABC. A general description was followed by three levels of information extraction: the number of cases included, the number of articles published per year, and the contributions of TDABC to achieve cost savings and other improvements.

**Results:**

Fourteen studies using real-world patient-level data to evaluate costs comprised the manual group of studies. Thirteen studies that reported the use of CM comprised the technology-based group of articles. In the manual studies, the average number of cases included per study was 160, while in the technology-based studies, the average number of cases included was 4,767. Technology-based studies, on average, have a more comprehensive impact than manual ones in providing accurate cost information from larger samples.

**Conclusion:**

TDABC studies supported by technologies such as CM register more cases, identify cost-saving opportunities, and are frequently used to support reimbursement strategies based on value. The findings suggest that using TDABC with the support of technology can increase healthcare value.

## Introduction

Improving the quality and accuracy of cost information is among the challenges actively administered by healthcare policymakers and leaders, motivated by the transition in payment models and pressure to reduce waste in the healthcare system (Najjar et al., 2017). Especially after the COVID-19 pandemic period, when healthcare systems were expected to prove their capability to deliver care with high efficiency, making cost information available has been recognized as elementary in the continuous search for more sustainable, equitable, and excellent healthcare systems.

One of the first steps to defining strategies that can result in excellent care with financial responsibility is to determine if there are enough resources with the quality or knowledge necessary to achieve excellence in care delivery and how much it costs. Microcosting analysis supported by the time-driven activity-based costing (TDABC) method has been identified as the gold standard in the search for accurate answers to these questions (Kaplan, 2014; Keel et al., 2017; da Silva Etges et al., 2020).

Since TDABC’s first applications in the healthcare field by Prof. Robert Kaplan (Kaplan, 2014), several projects worldwide have achieved favorable results measured in cost savings and value-increase opportunities, especially in the surgical field. Until 2020, systematic reviews evaluated applications of the method (Keel et al., 2017; da Silva Etges et al., 2020). Among the challenges reported in most studies was the difficulty in automating the data collection, scaling the method, and, consequently, moving from research to a digital solution that can be implemented in the hospital’s routines to guide managers in their decision-making processes about delivering care with higher efficiency.

The last few years were also marked by the explosion of health tech and by the consensus of the requirement to establish data-driven organizations in healthcare that can better use real-world evidence to guide effective health policies (Kraus et al., 2021; Ebbert et al., 2023). Among the solutions identified in published articles, the CareMeasurement software (Avant-garde Health, Boston, USA) (CM) makes a demonstrated contribution to some of the problems reported by the previous TDABC systematic reviews. It allows the automation of time stamps, resource consumption data collection, and the scalability of the TDABC as a routine to manage costs and has assisted managers in taking actions with a high likelihood of providing cost savings to hospital organizations (Carducci et al., 2020; Fang et al., 2021a; Carducci et al., 2021).

This study evaluated the influence of using technology on measuring accurate costs in healthcare organizations by identifying how such technology contributed to increasing value in the most recent scientific evidence of TDABC application in surgical pathways.

## Methods

This is a literature-based analysis that mainly used two data sources: the most recent systematic review that specifically evaluated TDABC studies in the surgical field, published in 2020, and all articles that mentioned the use of a CM to implement TDABC, which started to be published after 2020.

### Literature search strategy

PubMed was used to confirm the most recent systematic review, specifically exploring the use of TDABC in surgical pathways. Seven articles were found when searching for systematic reviews of TDABC on PubMed. The most recent, published in 2023, is specific for interventional radiology (Bulman et al., 2023) and spine surgeries (Ali et al., 2023). In 2022, a systematic review that evaluated the cost measure of value-based healthcare but did not specifically focus on TDABC studies was published (Leusder et al., 2022). In 2019 and 2018, studies specific to joint replacement (Pathak et al., 2019) and cancer (Alves et al., 2018) were published. The other two articles represent systematic reviews focused on evaluating TDABC in healthcare not associated with a specific clinical field or including other cost methods, the first published in 2017 (Keel et al., 2017) and the most recent in 2020 (da Silva Etges et al., 2020). This last one was used to identify the manual studies in this article.

The studies using CM were also retrieved from PubMed. The search was supported by the research team from the company responsible for CM development, Avant-garde Health, who organized the studies developed using data from the software that were indexed on Pubmed and used CM to extract or analyze cost information following the TDABC principles.

Both groups only considered original articles written in English.

### Data analyses

The articles from the systematic review (da Silva Etges et al., 2020) were grouped as manual, while those using CM were grouped as technology-based studies of TDABC implementation. The analysis compared the methodological aspects and accuracy of the results from both sets of articles and focused on evaluating the impact of using technology to apply TDABC. A general description, including the most frequent clinical fields and journals, was followed by three levels of information extraction: the number of cases included, the number of articles published per year, and the contributions of TDABC to achieve cost savings and redefine supply pricing and reimbursement strategies based on value.

For all microcosting articles included in the systematic review and in the group of CM articles, information on the number of cases included was extracted, and a mean number of articles that used manual methods or were supported by technology was calculated. Articles from the systematic review that were not based on a microcosting method and did not use a sample of patients were excluded from this analysis. For both groups, the number of articles published per year was computed, and the publication rates were compared.

A final analysis consisted of extracting the cost savings estimates achieved in manual and technology-based studies and contributions from the TDABC projects in redesigning more sustainable reimbursement programs. The mean cost savings were compared to evaluate the impact of technology on the hospital’s capabilities to increase its financial sustainability.

## Results

**FIGURE 1:**
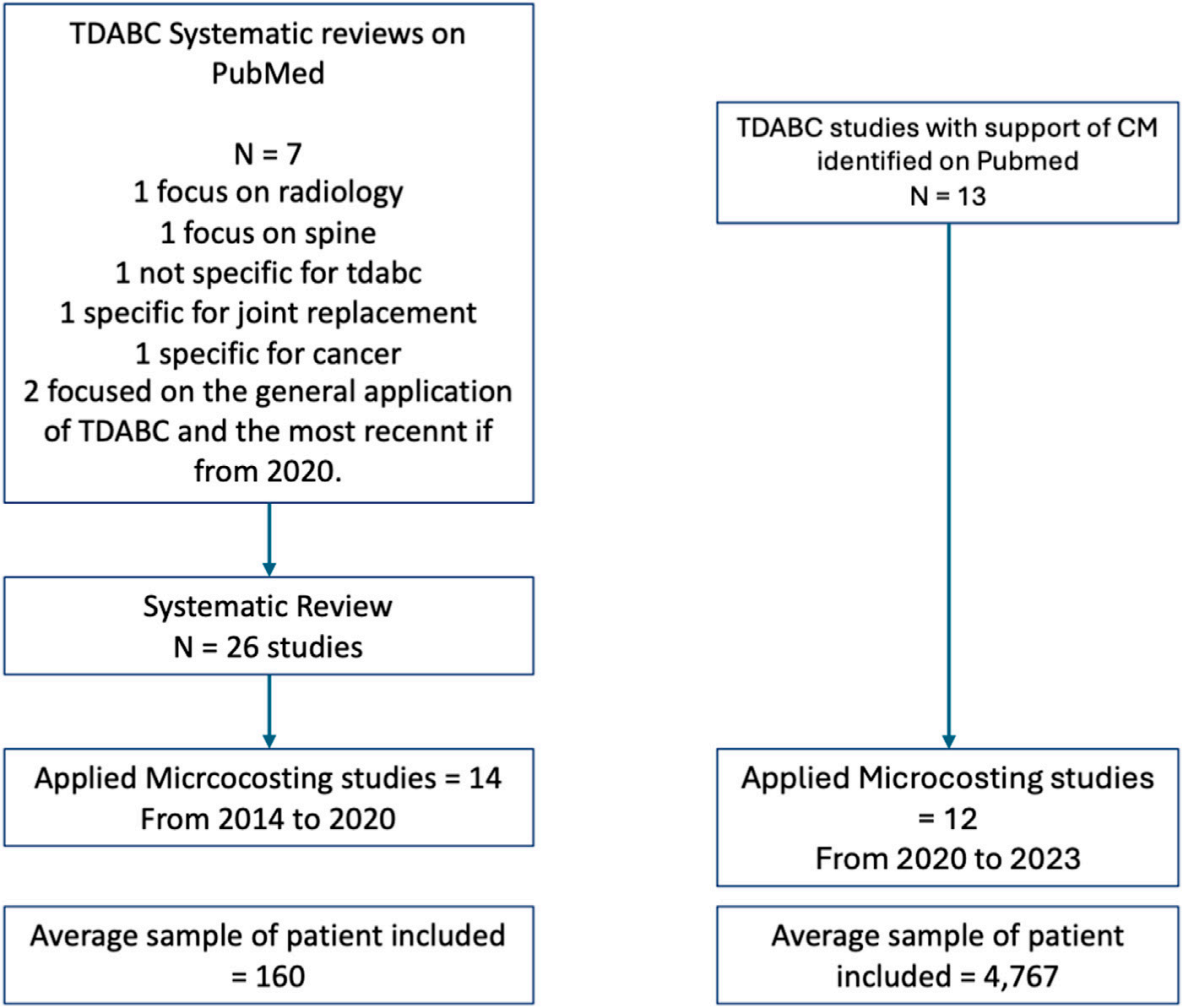
Flowchart of the studies included.

Among the 26 articles included in the systematic review, only 14 were applied microcosting studies that used real-world data at a patient level to evaluate costs. These studies comprised the manual studies. All studies that reported the use of CM were microcosting and applied studies and comprised the technology-based group of articles. In the manual group, the first evidence published is from 2014, and the group accounts for 14 studies published until 2020. In the technology-based group, 13 studies were identified that had been published in three years. Among the manual studies, the average number of cases included was 160, while among the technology-based studies, the average number of cases included was 4,767. Figure 1 contains the flowchart of the studies included, and Table 1 contains the articles included in both groups, the surgical fields, and the total number of patients included.

**TABLE 1:** Studies included in each group, surgical field, sample, and publication year.

Group	Study	Cost-saving result	Reimbursement or supply pricing contributions	Other TDABC contributions
Manual studies	Simmonds et al. (2019)	Estimated that 57% of the overhead costs attributed to the adenotonsillectomy procedures by the relative value units (RVU) system were from equipment and implants used by different hospital services	NA	NA
	Hamid et al. (2017)	Demonstrated how the TDABC can identify inefficiencies and result in cost savings. However, the study did not estimate or measure the potential cost savings that could be achieved in the surgery studied	NA	NA
	Koehler et al. (2019)	NA	NA	Compared technologies but did not explore potential cost savings in the same care pathway
	Odhiambo et al. (2019)	Estimated that the redundant staff members in the operating room represent an additional opportunity cost of £15 per minute, which represents, on average, a potential net loss of £1,000 per additional or delayed surgery hour	NA	NA
	Laviana et al. (2018)	NA	Compared TDABC results with traditional cost methods suggesting the value of this level of information to define accurate reimbursement strategies	NA
	Balakrishnan et al. (2018)	NA	NA	Compared technologies but did not explore potential cost savings in the same care pathway
	Chen et al. (2015)	Reduced duration and costs in the emergency department (−41 min, −$23) and preoperative floor (−57 min, −$18). Same-day discharge protocol eliminated postoperative floor costs (−$306). All three interventions reduced the total direct costs by 11% ($2753.39 to $2447.68) and the duration of hospitalization by 51%	NA	NA
	Husted et al. (2018)	NA	NA	Compared technologies but do not explore potential cost savings in the same care pathway
	McCreary et al. (2018)	NA	NA	Compared technologies but do not explore potential cost savings in the same care pathway
	Akhavan et al. (2016)	NA	Compared TDABC results with traditional cost methods suggesting the value of this level of information to define accurate reimbursement strategies	NA
	Yangyang et al. (2016)	NA	NA	Demonstrated how TDABC can identify inefficiencies and result in cost savings. However, did not estimate or measure the potential cost savings that could be achieved in the surgery studied
	McLaughlin et al. (2014)	NA	NA	Demonstrated how TDABC can identify inefficiencies and result in cost savings. However, did not estimate or measure the potential cost savings that could be achieved in the surgery studied
	Yangyang et al. (2017)	NA	NA	Compared technologies but did not explore potential cost savings in the same care pathway
	da Silva Etges et al. (2019)	NA	NA	Described the step-by-step process to execute TDABC as a microcosting technique
Technology-based studies	Carducci et al. (2021)	NA	Demonstrated the value of TDABC to make supply and labor costs transparent, suggesting that this level of transparency is necessary for the establishment of more accurate and profitable agreements with suppliers	NA
	Fang et al. (2021b)	Used accurate data from CM to evaluate the impact of implementing reference pricing (RP) for total knee arthroplasty supplies (TKS). Demonstrated that hospital costs for total knee arthroplasty (TKA) implants decreased by 16.7% after implementing RP. All the individual implant components decreased in costs	NA	NA
	Carducci et al. (2020)	NA	NA	The study focused on measuring the value index for total shoulder arthroplasty
	Carducci et al. (2020)	Determined that the implant is the most expensive cost item for all types of arthroplasties, identifying centers where it is possible to pay lower prices for implants that result in lower costs	NA	NA
	Chisari et al. (2021)	NA	Estimated the incremental cost of performing total knee arthroplasty (TKA) *versus* unicompartmental knee arthroplasty (UKA), arguing that the reimbursement for TKA should be reviewed to cover the incremental labor costs compared with UKA	NA
	Theosmy et al. (2021)	NA	Measured the differences between inpatient and outpatient TKA and compared them in terms of the reimbursement fees implemented for each type of surgery, arguing that by better adjusting the fee to the cost, it will be possible to economically benefit the healthcare system	NA
	Zachwieja et al. (2020)	Measured the potential profit increase of conducting overlapping surgeries. In the study, it was estimated to yield a potential profit increase of $1,215 per overlapping surgery in 8 h	NA	NA
	Yayac et al. (2020)	Determined that the increased cost of a cementless implant is recouped through savings in the cost of cement and supplies, as well as shorter operative times	NA	NA
	Goh et al. (2022)	Using TDABC, it was demonstrated that overall facility costs were lower in robot-assisted UKA (RA-UKA) despite a longer operative time. To facilitate wider adoption of this technology, implant manufacturers may negotiate lower implant costs based on volume commitments when robotic assistance is used. These supply cost savings appear to offset a portion of the increased costs	NA	NA
	Fang et al. (2021c)	NA	NA	Compared costs between the two groups but did not explore potential cost savings
	Fang et al. (2021d)	NA	NA	Compared costs between the two groups but did not explore potential cost savings
	Fang et al. (2021a)	NA	Demonstrated the value of TDABC to make supply and labor costs transparent, suggesting that this level of transparency is necessary for the establishment of more accurate and profitable agreements with suppliers	NA
	Fang et al. (2021e)	NA	Compared TDABC results with traditional cost methods, suggesting the value of this level of information to define accurate reimbursement strategies	NA

Since TDABC implementations began to receive technological support, the contributions in terms of cost savings estimates and the generation of accurate cost information to adjust reimbursement strategies have been more frequent. In the manual group, only two articles explored the use of costs based on TDABC to define reimbursement strategies at a macro level and compared TDABC with traditional methods but did not measure the impact of the differences in hospital sustainability (Akhavan et al., 2016; Laviana et al., 2018). In contrast, five studies from the technology-based group explored potential impacts on the definition of reimbursement strategies and were able to measure variabilities in cost items (labor, supply, medication) and between technologies or patient profiles (Fang et al., 2021a; Carducci et al., 2021; Chisari et al., 2021; Fang et al., 2021e; Theosmy et al., 2021). Comparing the cost information granularity between both groups reveals that the technology has potentialized the managers’ capabilities to identify the cost components responsible for the highest variabilities and, consequently, guide the efforts to adjust reimbursement strategies and deliver better care.

For cost-saving estimates, the differences observed are more concentrated in the cost variables explored and how to use them to estimate the potential economic impact at a hospital level. The manual studies focused on opportunities to reduce the length of time in the operating room and redesign surgical processes, resulting in suggestions to redefine hospital processes to reduce waste (Chen et al., 2015; Hamid et al., 2017; Odhiambo et al., 2019; Simmonds et al., 2019). The technology-based studies focused much more on variabilities and opportunities to renegotiate supply pricing and, because of the volume and cost proportions represented, estimate the important potential economic impact at the hospital and healthcare system levels (Fang et al., 2021a; Carducci et al., 2021; Chisari et al., 2021; Fang et al., 2021e; Theosmy et al., 2021). Supply cost-saving opportunities were not mentioned by the manual studies, and this seems to be where the studies that included more data encountered the highest cost-saving opportunities in surgeries that use high-cost supplies. Table 2 demonstrates how the studies from each group increased value by yielding cost savings, were used to sustain new reimbursement agreements, or explored other contributions from the TDABC.

**TABLE 2:** Contributions from applying TDABC to increase value.

Group	Title	Surgery field	Number of cases included	Year
Manual studies	Comparing the real and perceived cost of adenotonsillectomy using time-driven activity-based costing (Simmonds et al., 2019)	Adenotonsillectomy	53	2019
	Determining the cost-savings threshold and alignment accuracy of patient-specific instrumentation in total ankle replacements (Hamid et al., 2017)	Ankle replacement	87	2017
	Endoscopic versus open carpal tunnel release: A detailed analysis using time-driven activity-based costing at an academic medical center (Koehler et al., 2019)	Endoscopic vs. carpal tunnel release	40	2019
	Health facility cost of Cesarean delivery at a rural district hospital in Rwanda using time-driven activity-based costing (Odhiambo et al., 2019)	Cesarean	197	2019
	Retroperitoneal *versus* transperitoneal robotic-assisted laparoscopic partial nephrectomy: a matched-pair, bicenter analysis with cost comparison using time-driven activity-based costing (Laviana et al., 2018)	Retroperitoneal *versus* transperitoneal robotic-assisted laparoscopic partial nephrectomy	355	2018
	TDABC: Lessons from an application in healthcare (Balakrishnan et al., 2018)	Endoscopic vs. carpal tunnel release	180	2018
	Time-driven activity-based costing of total knee replacement surgery at a London teaching hospital (Chen et al., 2015)	Knee replacement	20	2015
	Time-driven activity-based cost of outpatient total hip and knee arthroplasty in different set-ups (Husted et al., 2018)	Hip and knee arthroplasty	6	2018
	Time-driven activity-based costing in fracture care: is this a more accurate way to prepare for alternative payment models? (McCreary et al., 2018)	Surgical treatment of isolated ankle fractures	35	2018
	Time-driven activity-based costing more accurately reflects costs in arthroplasty surgery (Akhavan et al., 2016)	Arthroplasty surgery	677	2016
	Time-driven activity-based costing to identify opportunities for cost reduction in pediatric appendectomy (Yangyang et al., 2016)	Appendicitis surgery	149	2016
	Time-driven activity-based costing: a driver for provider engagement in costing activities and redesign initiatives (McLaughlin et al., 2014)	Neurosurgery and urology	124	2014
	Time-driven activity-based costing: a dynamic value assessment model in pediatric appendicitis (Yangyang et al., 2017)	Pediatric appendicitis	208	2017
	An 8-step framework for implementing time-driven activity-based costing in healthcare studies (da Silva Etges et al., 2019)	Bone marrow transplant	12	2019
Technology-based studies	Identifying surgeon and institutional drivers of cost in total shoulder arthroplasty: a multicenter study (Carducci et al., 2021)	Shoulder arthroplasty	1.571	2020
	Reference pricing reduces total knee implant costs (Fang et al., 2021b)	Knee replacement	7.148	2020
	Variation in the value of total shoulder arthroplasty (Carducci et al., 2020)	Shoulder arthroplasty	239	2020
	Variation in the cost of care for different types of joint arthroplasty (Carducci et al., 2020)	Arthroplasty surgery	22.215	2020
	Despite equivalent Medicare reimbursement, facility costs for outpatient total knee arthroplasty are higher than unicompartmental knee arthroplasty (Chisari et al., 2021)	Knee replacement	2.641	2020
	Is the new outpatient prospective payment system classification for outpatient total knee arthroplasty appropriate? (Theosmy et al., 2021)	Knee replacement	4.496	2020
	Overlapping surgery increases operating room efficiency without adversely affecting outcomes in total hip and knee arthroplasty (Zachwieja et al., 2020)	Knee and hip replacement	4.786	2020
	The use of cementless components does not significantly increase procedural costs in total knee arthroplasty (Yayac et al., 2020)	Knee replacement	2.426	2020
	Robotic-assisted versus manual noncompartmental knee arthroplasty: a time-driven activity-based cost analysis (Goh et al., 2022)	Knee replacement	265	2022
	Differences in hospital costs among octogenarians and nonagenarians, following primary total joint arthroplasty (Fang et al., 2021c)	Arthroplasty surgery	889	2021
	Episode-of-care costs for revision total joint arthroplasty by decadal age groups (Fang et al., 2021d)	Arthroplasty surgery	551	2021
	Financial burden of revision hip and knee arthroplasty at an orthopedic specialty hospital: higher costs and unequal reimbursements (Fang et al., 2021a)	Knee and hip replacement	13.946	2021
	The cost of hip and knee revision arthroplasty by diagnosis-related groups: comparing time-driven activity-based costing and traditional accounting (Fang et al., 2021e)	Knee and hip replacement	793	2021

## Discussion

TDABC studies supported by technologies such as CM register more cases and deliver more precise measures to identify cost-saving opportunities, mainly based on supply variabilities. They are frequently used to redefine reimbursement strategies based on value, and potential improvements may be implemented more quickly. This suggests that the challenge of scaling the organizational capability to measure costs per care pathway at a patient level (Keel et al., 2017; da Silva Etges et al., 2020; Tsai et al., 2018) has started to receive answers from health tech companies. Healthcare leaders and policymakers should take note of how these advances impact the precision of cost information and its use as real-world data in health technology assessment processes, the continuous effort to reduce waste in healthcare, and the acceleration of implementing data-driven value-based reimbursement.

In his seminal book on health economics (Drummond et al., 2005), Prof. Michael Drummond pointed out microcosting techniques as the best strategies to provide accurate cost information for use in economic models to guide health policies and HTA processes. Several researchers have agreed that microcosting is the only way to understand and measure how each individual with a specific clinical condition is consuming resources from the healthcare system. It is not pricing, charges, or fee analyses; microcosting measures resource consumption, which should be used as a parameter to define more assertive reimbursement strategies adjusted to outcomes and clinical conditions (Tan et al., 2009). TDABC is an effective method for performing microcosting analysis (da Silva Etges et al., 2019). However, for the health economics community, the bottleneck from microcosting techniques is the capability to generate representative cost information from a population due to the complexity of data collection and analysis (Drummond et al., 2005). In an era where each day, more uses of real-world financial and clinical data are emerging and being recommended by the reglementary agencies, such as the FDA and NICE (Sherman et al., 2016; Jarow et al., 2017), high data accuracy and difficulty to scale and generate representative information represents a trade-off that deserves answers. By consolidating evidence from technology-based studies that incorporate a larger number of cases and detailed cost information, especially regarding supply consumption, this review provides a crucial starting point for implementing data-driven strategies to reduce waste, improve population health, and increase value.

The next step in affecting people’s lives through improved data quality involves valuing “health” rather than “healthcare service delivered” by redefining reimbursement strategies, such as strategies based on value (Porter and Kaplan, 2016). The success of implementing value reimbursement strategies relies on the level of granularity in outcomes and cost data that stakeholders can monitor, including specific details related to patients’ consumption patterns based on their clinical condition. Achieving this level of granularity requires using technology that enables the ethical and compliant sharing of data. The technology-based studies that contributed to the definition of reimbursement strategies reported how CM allowed making supply and labor costs transparent on a very detailed level. It was noted as a significant advance achieved by technology and a differential to define the agreements involving the device industry and payers. All these initiatives are recent and are in a proof-of-concept period in most countries, with a consensus that having good-quality data is a requirement for establishing effective agreements (Agarwal et al., 2020).

In the orthopedic field, implants comprise approximately 50% of surgical costs, with revision surgeries being more expensive than primary procedures (Fang et al., 2021a). Pricing strategies with suppliers have been developed to reduce costs during hospital surgical processes (Collins et al., 2017). Reliable pricing strategies require accurate and transparent data, which can be obtained using software, such as CM, that provides real-time, detailed information about individual consumption, enabling the control of payments between stakeholders.

Limitations: Although this study is innovative in its evaluation of the impact of technological advancements on measuring healthcare costs and defining reimbursement and supply pricing agreements, there are some limitations to consider. The analysis presented focused on evaluating the advances based on one disseminated technology to scale TDABC analysis in the healthcare field. Future research could use the results reported to examine emerging technologies. It is expected that the capability to compare solutions and identify benchmarks for making healthcare more effective and data-driven will improve with the advancement of digital technologies. In this future scenario, having previous review studies, such as this one, can accelerate the process of identifying evidence from specific solutions available on the market. Additionally, the cases identified in the technology-based group are from the United States, and further research is needed to evaluate the variability of the impacts of redefined agreements in different cultural contexts. Finally, this study only focuses on surgical pathways, and there is a gap in the literature regarding the implications for clinical pathways.

## Conclusion

TDABC studies supported by technologies such as CM register more cases, identify cost-saving opportunities, mainly based on supply variabilities, and are frequently used to redefine reimbursement strategies based on value. Our findings suggest that using TDABC with the support of technology can accelerate the process of redefining payment agreements with suppliers and, consequently, healthcare payers, contributing to reducing waste and establishing a more financially adjusted and value-based healthcare system.

## Summary points

- TDABC studies supported by technologies such as CM are registering more cases.
- Technology advances are contributing to delivering more precise measures to identify cost-saving opportunities, mainly based on supply variabilities.
- Healthcare leaders are using these advances to redefine reimbursement strategies based on value.
- TDABC, with the support of technology, can serve as a solid element to accelerate the process of redefining payment agreements with suppliers and, consequently, healthcare payers. It contributes to reducing waste and establishing a more financially adjusted and value-based healthcare system.
